# TRPM4 is highly expressed in human colorectal tumor buds and contributes to proliferation, cell cycle, and invasion of colorectal cancer cells

**DOI:** 10.1002/1878-0261.12566

**Published:** 2019-09-12

**Authors:** Sven Kappel, Paulina Stokłosa, Barbara Hauert, Daniela Ross‐Kaschitza, Anna Borgström, Roland Baur, José A. Galván, Inti Zlobec, Christine Peinelt

**Affiliations:** ^1^ Institute of Biochemistry and Molecular Medicine University of Bern Switzerland; ^2^ Graduate School for Cellular and Biomedical Sciences University of Bern Switzerland; ^3^ Institute of Pathology Translational Research Unit University of Bern Switzerland

**Keywords:** cancer hallmarks, cell cycle analysis, colorectal cancer, CRISPR/cas9, TRPM4, tumor buds

## Abstract

Transient receptor potential melastatin‐4 channel (TRPM4) dysregulation contributes to heart conditions, immune diseases, and cervical and prostate cancer. Up to now, the involvement of TRPM4 in colorectal cancer (CRC) pathophysiology remains unknown. Here, we investigated tumor tissue microarrays from 379 CRC patients and analyzed TRPM4 protein expression, tumor characteristics, and clinical outcome. High TRPM4 protein expression was associated with unfavorable tumor features characteristic for epithelial–mesenchymal transition and infiltrative growth patterns, that is, a high number of tumor buds and a low percentage in tumor border configuration. Compared to CRC cells representing early cancer stages, TRPM4 protein expression was the highest in cells representing late‐stage metastatic cancer. Investigation of CRC cell line HCT116 and five CRISPR/cas9 TRPM4 knockout clones demonstrated that TRPM4 exhibited large Na^+^ current densities (~ 60 pA/pF). In addition, CRISPR/cas9 TRPM4 knockout clones showed a tendency toward decreased migration and invasion, cell viability, and proliferation and exhibited a shift in cell cycle when compared to HCT116. Stable overexpression of TRPM4 (TRPM4 wild‐type) in two CRISPR/cas9 TRPM4 knockout clones rescued the decrease in cell viability and cell cycle shift. Stable overexpression of a nonconducting, dominant‐negative TRPM4 mutant (TRPM4 D894A) did not rescue the decrease in viability or cell cycle shift. Taken together, these findings pointed to TRPM4 ion channel conductivity as the underlying mechanism for decreased viability and cell cycle shift in the TRPM4 knockout clones. Together with previous findings, our present data suggest that TRPM4 plays a versatile role in cancer cell proliferation, cell cycle, and invasion.

**Abbreviations**
CD: current density
CRC: colorectal cancer
TBC: tumor border configuration
TRPM4: transient receptor potential melastatin‐4 channel

## 1 Introduction

In 2018, 1.8 million cases of colorectal cancer (CRC) were registered worldwide and ~ 881 000 patients died of CRC (World Health Organization, 2018). As in other types of cancer, driver mutations in several key signaling pathways promote CRC pathogenicity. In particular, alterations in the PI3K, WNT, and KRAS pathways lead to increased migration and proliferation of CRC cells (Dienstmann *et al.*, 2017; Huang *et al.*, 2018). Emerging evidence reveals that ion channel malfunctions within these pathways contribute to cancer hallmark functions (Bose *et al.*, 2015; Litan and Langhans, 2015).

Transient receptor potential melastatin‐4 channel (TRPM4) is expressed in several human tissues (Launay *et al.*, 2002; Nilius *et al.*, 2003) and has been identified as a cancer driver gene that contributes to migration, proliferation, and invasion of prostate cancer (PCa) cells (Berg *et al.*, 2016; Holzmann *et al.*, 2015; Sagredo *et al.*, 2018, 2019; Schinke *et al.*, 2014). In PCa, TRPM4 expression is negatively regulated by microRNA‐150, which results in a shift in cell cycle to G1 phase (Hong and Yu, 2018). The mechanism of TRPM4’s contribution to PCa hallmark functions remains unclear. It may be based on altered Ca^2+^ signaling, since Na^+^ influx *via* TRPM4 can reduce the driving force for Ca^2+^ entry (Barbet *et al.*, 2008; Cheng *et al.*, 2007; Holzmann *et al.*, 2015; Kilch *et al.*, 2016; Shimizu *et al.*, 2009). Other reported mechanisms of action for TRPM4 include WNT pathway alterations, interactions of TRPM4 with other proteins, and localization of TRPM4 in the focal adhesome (Blanco *et al.*, 2019; Cáceres *et al.*, 2015; Gerzanich *et al.*, 2018; Sagredo *et al.*, 2019). In prostate and cervical cancer cells, TRPM4 contributes to proliferation by decreasing Akt/GSK3‐β activity and enhancing β‐catenin signaling in the WNT pathway (Armisén *et al.*, 2011; Sagredo *et al.*, 2018). In contrast, public database analysis identified TRPM4, together with six other differentially expressed genes, as a good prognostic marker for endometrial cancer (Liu *et al.*, 2019). In CRC, TRPM4 mRNA expression is reported to be decreased (Sozucan *et al.*, 2015) or not changed (Pérez‐Riesgo *et al.*, 2017). It has also been suggested that TRPM4 may play a role in Ca^2+^‐induced mucin secretion from goblet cells (Cantero‐Recasens *et al.*, 2019). The role of TRPM4 in CRC pathophysiology has not been investigated.

Given the heterogeneous nature of cancerous tissues, our study focuses on TRPM4 protein expression in CRC tissue instead of TRPM4 mRNA expression. In the present study, we analyzed TRPM4 protein expression levels in CRC tissue from 379 patients and examined its correlation with clinical and tumor parameters. Furthermore, we investigated TRPM4’s contribution to CRC cell proliferation, viability, migration, invasion, cell cycle, and TRPM4 ion conductivity as the possible underlying mechanism.

## 2 Materials and Methods

### 2.1 Patients and tissue microarray construction

This study included a retrospective collective of 379 patients who were diagnosed with primary CRC at the Institute of Pathology, University of Bern, between 2002 and 2013. Table S1 presents the patient characteristics. No patients received preoperative therapy. Information regarding overall survival and postoperative therapy was available for 378 patients. CRC samples were mounted onto next‐generation Tissue Microarrays (ngTMA^®^, Zlobec *et al.*, 2013). From all corresponding tissue blocks, H&E‐stained sections were scanned and digitally annotated using a designated TMA annotation tool. Multiple areas were marked, including regions from the tumor center (TC), tumor invasion front, and tumor microenvironment (TME). An average of 6–8 punches per CRC was taken. The use of tissues and data in this study was approved by the local ethics committee (Ref: 200/2014). This study was conducted in accordance with the guidelines set by the Declaration of Helsinki.

### 2.2 Immunohistochemistry

All ngTMA^®^ blocks were cut into 2.5‐µm sections and dried at 60 °C for 30 min. Immunohistochemistry was performed using an automated immunostainer (Leica Bond RX, Leica Biosystems, Nussloch, Germany). After deparaffinization, antigen retrieval was performed in citrate buffer at 100 °C for 30 min. Then, endogenous peroxidase was blocked with H_2_O_2_ for 5 min. Samples were incubated with TRPM4 rabbit antibody (1 : 200 dilution; generated by Pineda, Ozhathil *et al.*, 2018) for 30 min and then with horseradish peroxidase‐polymer for 15 min. Visualization was performed using 3,3‐diaminobenzidine as a brown chromogen (Bond Polymer Refine Detection Kit, Leica Biosystems, Ref DS9800) for 10 min. Finally, hematoxylin was applied for 5 min to counterstain nuclei. Slides were scanned with Pannoramic 250 Flash II and photographed in CaseViewer (3DHistech, Budapest, Hungary). Staining was homogeneous from punch to punch; thus, the intensity of each core was evaluated and scored as either negative (no epithelium showed immunoreactivity for the protein), 1 (weak intensity), or 2 (strong intensity). Each tissue location was scored independently [TC, tumor front (TF), and TME]. In tissues with more than one punch per patient, median values were included in the statistical analysis. Descriptive statistics were used for TRPM4. Chi‐square test was performed to determine the association of expression with categorical variables (Table S1), and a nonparametric Kruskal–Wallis test was used for continuous variables. All *P* values are two‐sided, and *P* < 0.05 was considered statistically significant. Analyses were performed using sas V9.2 (The SAS Institute, Cary, NC, USA) and graphpad prism 8 (GraphPad Software, San Diego, CA, USA).

### 2.3 Cell culture

All cell lines were cultured according to ATCC (Manassas, VA, USA) guidelines. Standard growth medium supplemented with 0.25 μg·mL^−1^ puromycin (Gibco, Thermo Fisher, Waltham, MA, USA) was used for cultivation of cells with stable re‐expression of TRPM4 wild‐type (WT), TRPM4 D984A, and GFP control construct.

### 2.4 Generation of HCT116 TRPM4 knockout clones 1–5 by CRISPR/‐Cas9 technique

Two different deletions were generated: deletion 1 (9744 bp, chromosome 19: 95 975–105 719) between exons 2 and 4, and deletion 2 (454 bp, chromosome 19: 105 706–106 159) between exons 1 and 2. For each deletion, two‐guide RNAs were designed: gRNA, gRNA4.1/gRNA4.2 for deletion 1 and gRNA50fw/crRNA4 for deletion 2 (Heigwer *et al.*, 2014). The sense and antisense oligonucleotides for the gRNAs (Table S2) were cloned into a pSpCas9(BB)‐2A‐GFP vector (#48138; Addgene, Watertown, MA, USA) or a pU6‐(BbsI)‐CBh‐Cas9‐T2A‐mCherry vector (#64324; Addgene). Cloning was performed as previously described (ZhangLab). Transfection of HCT116 cells was performed with the 4D Nucleofector (Lonza, Basel, Switzerland) following the manufacturer's instructions. 2 × 10^6^ cells were transfected with 2 µg of each gRNA. At 24 h post‐transfection, cells were sorted for GFP/mCherry with ASTRIOS flow cytometry sorter (Beckman Coulter, Indianapolis, IN, USA). Clones were screened by genotyping. Primers and primer pairs are listed in Tables S3 and S4. Five of the 145 clones tested showed the desired deletion in both alleles (KO 1–4 deletion 1, KO 5 deletion 2).

### 2.5 Stable re‐expression of TRPM4 WT and TRPM4 D984A in TRPM4 KO 1–2 cells

Transient receptor potential melastatin‐4 WT, dominant‐negative TRPM4 (D984A), and GFP control constructs were amplified from TRPM4‐pMaxGFP and TRPM4D984A‐pMaxGFP vectors with primers listed in Table S5 and S6. Primers were designed according to ClonExpress^®^ Entry One Step Cloning Kit’s (Vazyme, Nanjing, China) instruction. The upstream recombination sequence and the downstream recombination sequence were designed to overlap with the piggyBac vector (5753 bp) cut with BamHI and XhoI. The recombination reaction was performed with ClonExpress^®^ Entry One Step Cloning Kit, according to manufacturer’s guidelines. Transfection of TRPM4 WT, TRPM4 D984A, and control vector was performed in KO 1 and 2 cells. Cells were cotransfected with corresponding piggyBac vector and transposase vector with the use of FuGENE transfection kit (Promega, Madison, WI, USA). For selection, cell culture media was changed to medium supplemented with 0.5 μg·mL^−1^ puromycin (Gibco) 24–48 h after transfection. After 1–2 weeks, all cells were positively transfected (green fluorescence). To achieve similar levels of expression and remove cells with highly overexpressed TRPM4, cells were further sorted for GFP intensity with the ASTRIOS flow cytometry sorter. Only cells showing low fluorescence were used for further experiments.

### 2.6 Quantitative real‐time PCR

Total RNA was isolated from HCT116 cell pellets using the QIAshredder and the RNeasy Mini kit (Qiagen, Hilden, Germany). Reverse transcription was performed with 2 µg RNA and the High‐Capacity cDNA Reverse Transcription Kit (Thermo Fisher). Diluted cDNA (1 : 4) was used for qPCR with the TaqMan Gene Expression Assay (Thermo Fisher). PCR conditions were as follows: 2‐min activation at 50 °C, then 10 min at 95 °C; followed by 40 cycles of 15‐s denaturation at 95 °C and 1‐min annealing at 60 °C. Expression levels of target genes were normalized to expression of the reference genes RNA polymerase II (RNAPol II, Hs00172187_m1) and TATA‐binding protein (TBP, Hs00427621_m1). The detected target sequences were TRPM4 Hs00214167_m1 (spans exons 12/13) and TRPM4 Hs01026061_m1 (spans exons 3/4).

### 2.7 Western blot analysis

Total protein lysate (50 µg) was separated on 10% SDS/PAGE, and proteins were probed using rabbit TRPM4 antibody (1 : 500; generated by Pineda, Ozhathil *et al.*, 2018) and mouse β‐actin antibody (1 : 2000; Cell Signaling Technology, Leiden, The Netherlands, #3700). Antibodies and proteins were detected with the LI‐COR (LI‐COR Biosciences, Lincoln, NE, USA) Odyssey Imaging System. Fluorescence of the secondary antibodies IRDye^®^ Donkey anti‐Mouse (#925‐68022; LI‐COR) and IRDye^®^ 800CW Goat anti‐Rabbit (#925‐32211; LI‐COR) was quantified using LI‐COR Image Studio Lite software. TRPM4 expression was normalized to β‐actin. Where applicable, statistical significance was analyzed with one‐way ANOVA in graphpad prism 8.

### 2.8 Electrophysiology

Patch‐clamp experiments were performed in a tight‐seal whole‐cell configuration at 22–25 °C. Currents were acquired with a HEKA EPC‐10 amplifier, digitized, and recorded with HEKA patchmaster v2x53 (HEKA, Lambrecht, Germany). Voltage ramps of 50‐ms duration, spanning −100 to +100 mV from a holding potential of 0 mV, were delivered every 2 s over a 240‐s period. All voltages were corrected for a 10‐mV liquid junction potential. Capacitive currents were determined and corrected before each voltage ramp delivery. Currents were filtered at 1 kHz and then sampled at 3 kHz. For analysis, currents were extracted at −80 and 80 mV, normalized to cell capacitance and plotted versus time. Data were analyzed with igor pro 6.37 (Wavemetrics, Lake Oswego, OR, USA). Bath solutions contained 140 mm NaCl, 0.5 mm CaCl_2_, 3 mm MgCl_2_, and 10 mm HEPES. In the *N*‐methyl‐d‐glucamine (NMDG) bath solution, 140 mm NaCl was replaced by 140 mm NMDG. pH was adjusted to 7.2 with NaOH or HCl. Osmolarity was adjusted to ~ 300 mOsm with glucose. Internal solution contained 140 mm Cs‐glutamate, 10 mm EDTA, 10 mm HEPES, and 8 mm NaCl. Concentrations of MgCl_2_ (3 mm) and CaCl_2_ (10 µm) were adjusted according to WEBMAXC STANDARD. Under these conditions, the patch pipettes resistances were between 2 and 3 MΩ.

### 2.9 Cell viability

Cells were seeded in triplicates on 96‐well plates at 1 × 10^4^ cells per well in standard growth media. Assays were performed using the RealTime‐Glo MT Cell Viability Assay kit (#G9711; Promega) according to the manufacturer’s instructions. Luminescence intensity was detected every hour over a 72‐h period using a Tecan Spark™ (Tecan, Männedorf, Switzerland) 10 m multimode microplate reader. For analysis, data for each measurement were normalized to the first data point. Statistical significance was analyzed using a Friedman test and Dunn’s test for multiple comparisons.

### 2.10 5‐Fluorouracil (5‐FU) viability assay

Cells were seeded in 96‐well plates at 1 × 10^4^ cells per well in standard growth media. After ~ 6 h, cell culture medium was changed to standard growth medium with NanoLuc Luciferase and MT Cell Viability Substrate (Promega). To induce cell death, cells were treated with 10 or 50 µm 5‐FU (#F6627; Sigma Aldrich, St. Louis, MO, USA). Measurements with a final concentration of 0.02% DMSO were used as a control. Cell viability was monitored over a 72‐h period using a Tecan Spark™ 10 m multimode microplate reader.

### 2.11 Proliferation

Proliferation was determined on xCELLigence^®^ (Acea Biosciences, San Diego, CA, USA) E‐Plates in an xCELLigence^®^ RTCA DP system. 4 × 10^4^ cells were plated in standard growth medium and proliferation was measured for 48 h in 15‐min intervals. Statistical significance was analyzed with Friedman test and Dunn’s test for multiple comparisons.

### 2.12 Cell cycle analysis

To determine cell cycle distribution, propidium iodide (PI; Abcam, Cambridge, UK) staining was performed according to the manufacturer’s guidelines. PI staining was detected with a BD LSR II BD flow cytometer and facsdiva software (BD Biosciences, Franklin Lakes, NJ, USA). Data analysis and statistical analysis (RM one‐way ANOVA and Holm–Sidak’s multiple comparison test) were performed using flowjo10 (FlowJo, LLC, Franklin Lakes, NJ, USA) and graphpad prism.

### 2.13 Migration and invasion

Migration and invasion assays were performed on xCELLigence^®^ CIM‐Plates in an xCELLigence^®^ RTCA DP device according to the manufacturer’s instructions. 4 × 10^4^ cells/100 µL in serum‐free medium were seeded, and migration toward medium containing 1 % FBS was measured at 15‐min intervals for 48 h. To measure invasion, an additional Corning^®^ Matrigel^®^ (LOT #7345017, 400 µg·mL^−1^) coating and medium containing 10 % FBS as invasion stimulus were used. Invasion data have been set to zero by subtraction of the cell index value at 24 h. Statistical significance was analyzed by Friedman test and Dunn’s multiple comparison test.

## 3 Results

### 3.1 Expression of TRPM4 in human CRC

**Figure 1:**
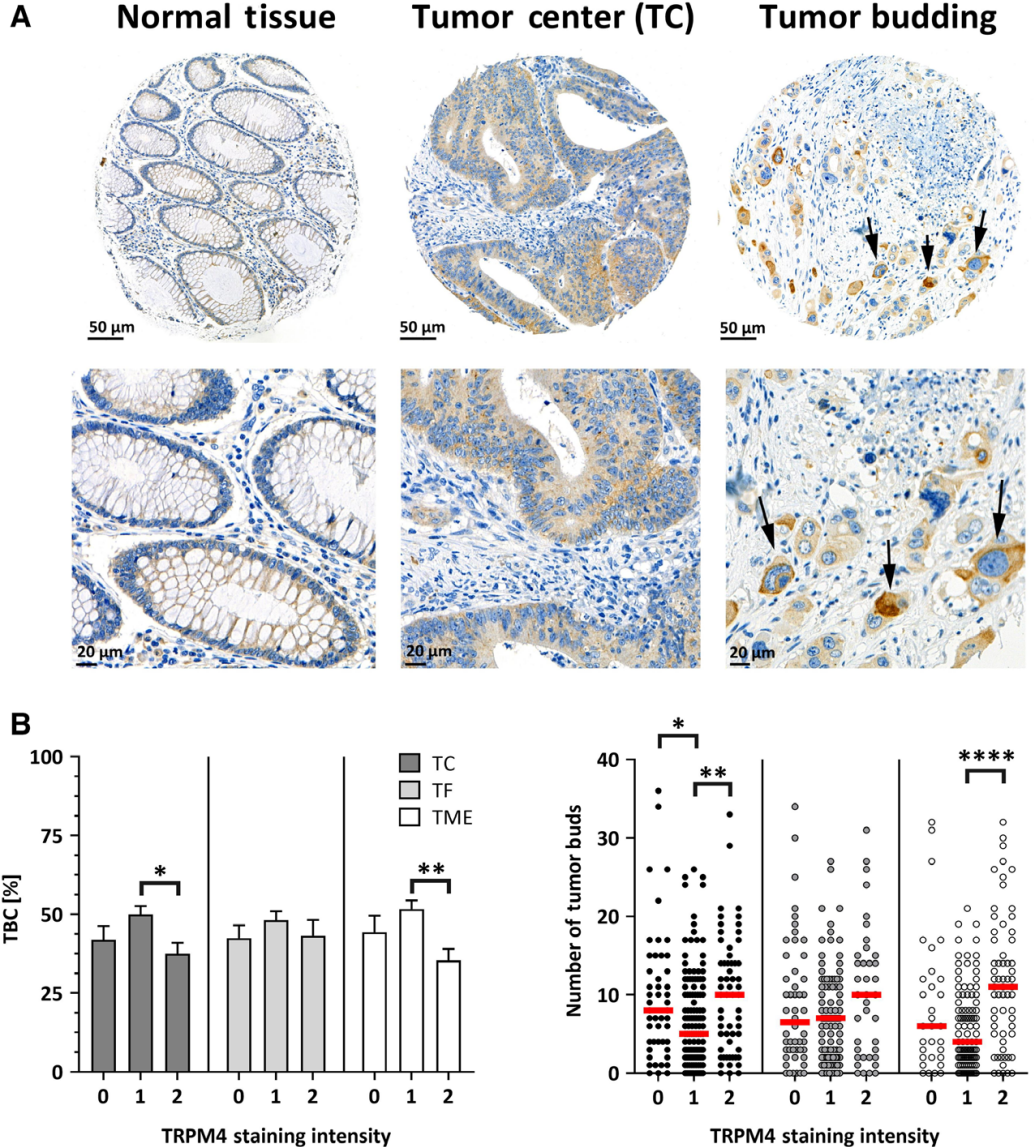
TRPM4 in human CRC tissue. (A) Overview (upper panels) and close‐up view (lower panels) of TRPM4 antibody staining of normal tissue (left panels), tumor tissue (middle panels), and TME (right panels). Arrows indicate tumor buds in the TME (right panels). Upper part: 20× magnification, 50‐µm scale bar. Lower part: 40× magnification, 20‐µm scale bar. (B) Bar diagram (mean + SEM) of TBC ratio in % plotted against TRPM4 staining intensities (*I*; negative, 0; weak, 1; strong, 2) from TC, TF, and TME. Numbers of biologically independent replicates (*n*) are *n* = 56 for *I* = 0, *n* = 168 for *I* = 1, and *n* = 81 for *I* = 2 in TC; *n* = 64 for *I* = 0, *n* = 134 for *I* = 1, and *n* = 48 for *I* = 2 in TF; and *n* = 43 for *I* = 0, *n* = 133 for *I* = 1, and *n* = 75 for *I* = 2 in TME. (C) Scatter plot of the numbers of tumor buds plotted against TRPM4 staining intensities. Numbers of biologically independent replicates are *n* = 50 for *I* = 0, *n* = 103 for *I* = 1, and *n* = 38 for *I* = 2 in TF; *n* = 48 for *I* = 0, *n* = 134 for *I* = 1, and *n* = 59 for *I* = 2 in TC; and *n* = 31 for *I* = 0, *n* = 102 for *I* = 1, and *n* = 62 for *I* = 2 in TME. Red bars represent medians. Asterisks indicate statistically significant differences analyzed by a Kruskal–Wallis test (*P < 0.05, **P < 0.005, ****P < 0.0001) in B and C.

Human normal and CRC tissue samples were stained with a TRPM4‐specific antibody in next‐generation Tissue Microarrays (ngTMA^®^; Fig. 1A). Figure S1 shows the specificity of the TRPM4 antibody: TRPM4 staining was detected in HCT116 cells, but not in HCT116 TRPM4 knockout cells when cells were fixed and stained using the same protocol as for the ngTMA. TRPM4 staining intensities in the TC and TF regions of CRC tissue, and in the TME, were digitally recorded. Staining intensities were rated as negative (0), weak (1), or strong (2). Tumor characteristics and clinical parameters were statistically analyzed according to TRPM4 staining intensity (Table S1).

The heterogeneous front of CRC tissue is characterized by areas with pushing or expanding tumor borders, and invasion sites exhibiting an infiltrative growth pattern and disseminated cells. The tumor border configuration (TBC) parameter was defined as the percentage of the pushing/infiltrating margin ratio. In CRC, low TBC percentages are associated with tumor invasiveness and higher tumor grades, lymphatic and vascular invasion, shorter patient survival, and an increased probability of lymph node metastasis (Karamitopoulou *et al.*, 2015). Figure 1B shows TBC analysis according to TRPM4 staining intensity from a Kruskal–Wallis test for nonparametric data. Strong TRPM4 staining intensities (score of 2) were associated with significantly lower TBC values (for TC and TME staining), indicating that high TRPM4 protein expression was associated with infiltrative growth patterns.

Tumor budding is defined as the presence of disseminated single CRC cells or small clusters of up to five CRC cells, which are often found in the TME with infiltrative growth patterns. CRC tumor budding is commonly associated with invasive tumor growth, epithelial–mesenchymal transition (EMT), and lymphovascular invasion (Georges *et al.*, 2018; Koelzer *et al.*, 2016). CRC tissue characterized by higher TRPM4 staining intensity was associated with significantly more tumor buds in the TME (Fig. 1C).

Overall, high TRPM4 protein expression levels correlated with a high number of tumor buds, and an infiltrative growth pattern (low percentage of TBC). Both characteristics are associated with tumor invasion and metastasis.

### 3.2 Expression of TRPM4 in CRC cells

While tumor nodes metastasis staging is the most common classification system for CRC, the Dukes system can be used to classify CRC into four categories. In Dukes stage A, cancer cells have invaded surrounding tissues, but the tumor has not yet penetrated the bowel wall. In Dukes stage B, cancer cells have invaded the muscle layer by breaking through the bowel wall, but lymph nodes are not involved. Dukes stage C indicates additional involvement of the lymph nodes, and Dukes stage D indicates CRC showing widespread metastasis.

**Figure 2:**
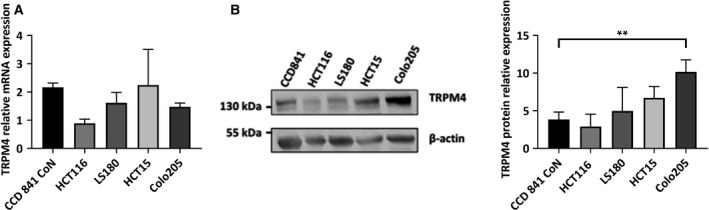
TRPM4 expression in CRC cells representing Dukes stages A–D. (A) TRPM4 mRNA levels in CCD 841 CoN (normal colon cells), HCT116 (Dukes stage A), LS180 (Dukes stage B), HCT15 (Dukes stage C), and Colo205 (Dukes stage D) normalized to TBP. The results from three independent experiments are presented as mean + SEM. (B) Representative western blot from CCD 841 CoN, HCT116, LS180, HCT15, and Colo205 cells (left panel). TRPM4 was detected with an anti‐TRPM4 antibody. β‐Actin was used as loading control and probed with an anti‐β–actin antibody. Quantification of protein expression levels shown as mean + SEM in CCD 841 CoN, HCT116, LS180, HCT15, and Colo205 cells analyzed from three independent experiments (right panel). TRPM4 protein expression is normalized to expression of β‐actin. Statistical significance was analyzed with one‐way ANOVA (**P < 0.005).

Here, we investigated TRPM4 expression in a colon cell line (CCD 841 CoN) and cancer cell lines representing CRC in Dukes stage A (HCT116), B (LS180), C (HCT15), and D (Colo205). In these cell lines, TRPM4 mRNA levels were determined by qPCR and normalized to two reference genes: TBP (Fig. 2A) and RNA Pol II (data not shown). Figure 2B (left panel) shows a representative western blot for TRPM4 protein expression in the different cell lines (normal cells and Dukes stage A–D). TRPM4 was normalized to β‐actin. Figure 2B (right panel) shows the average densitometry from three independent experiments. TRPM4 expression was elevated in the Dukes stages B, C, and D cell lines compared to normal cells, and this elevation was significant in Dukes stage D CRC cells.

### 3.3 Functional analysis of TRPM4 in HCT116 and HCT116 TRPM4 knockout cells

**Figure 3:**
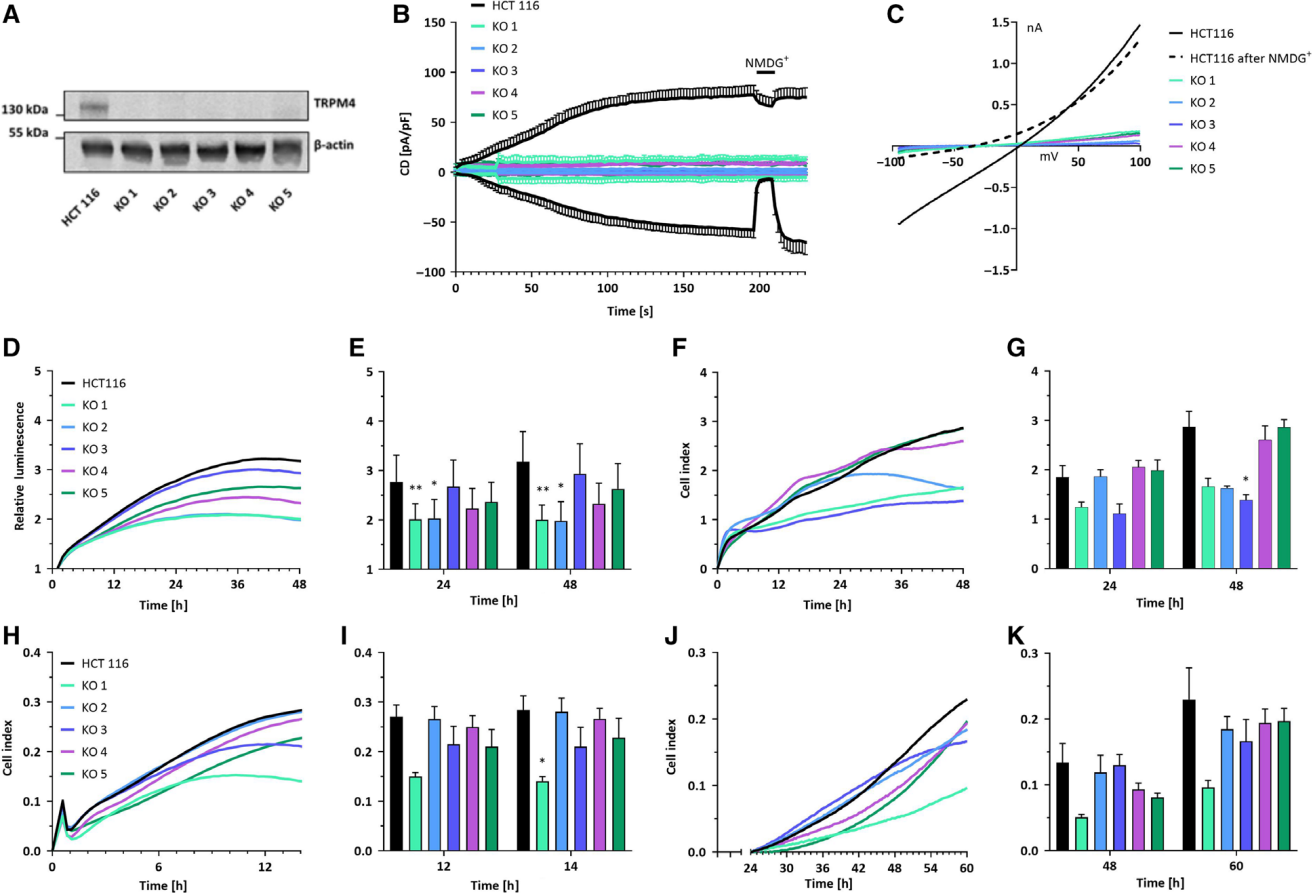
TRPM4 knockout (KO) and cancer hallmark functions in HCT116. (A) Western blot analysis from HCT116 and KO 1–5. Protein expression levels were detected using anti‐TRPM4 and anti‐β‐actin. (B) *I*_CAN_ was evoked with 10 µm Ca^2+^ in the patch pipette. Data are shown as mean ± SEM and numbers of biologically independent replicates are *n* = 6 for HCT116, *n* = 4 for KO 1, *n* = 4 for KO 2, *n* = 3 for KO 3, *n* = 3 for KO 4, and *n* = 3 for KO 5. (C) Corresponding IVs at 196 s from cells in B. (D) Cell viability was evaluated using a RealTime‐Glo MT assay. Mean of relative luminescence was plotted versus time. Three independent experiments were performed. (E) Bar diagram of data (mean + SEM) at 24 h and 48 h from the experiments in D. (F) Cell proliferation was determined with an xCELLigence^®^ system. Mean of cell index was plotted versus time. Three independent experiments were performed. (G) Bar diagram of data (mean + SEM) at 24 and 48 h from the experiment in F. (H) Mean of cell index of HCT116 cells and KO 1–5 in a migration assay plotted versus time. Each curve represents an average of at least three independent experiments (*n* = 9 for HCT116, *n* = 4 for KO 1, *n* = 5 for KO 2, *n* = 7 for KO 3, *n* = 5 for KO 4, and *n* = 7 for KO 5). (I) Bar diagram of data (mean + SEM) extracted at 12 h and 14 h from the experiments in H. (J) Mean of cell index of HCT116 cells and KO 1–5 in an invasion assay plotted versus time. Each curve represents an average of at least three independent experiments (*n* = 9 for HCT116, *n* = 4 for KO 1, *n* = 5 for KO 2, *n* = 7 for KO 3, *n* = 5 for KO 4, and *n* = 7 for KO 5). (K) Bar diagram of data (mean + SEM) extracted at 48 h and 60 h from experiments in J. Statistical analysis was performed using Friedman’s test with Dunn’s multiple comparison (*P < 0.05, **P < 0.005) in E, G, I, and K.

We generated five different HCT116 TRPM4 knockout clones (KO 1–5) with CRISPR/cas9 technique. Analysis by qPCR and western blot confirmed the absence of TRPM4 mRNA and protein expression (Fig. S2 and Fig. 3A). We assessed TRPM4 ion channel activity by whole‐cell patch‐clamp technique and found that 10 µm Ca^2+^ in the patch pipette activated *I*_CAN_ (Ca^2+^‐activated nonselective current) in HCT116. Currents at −80 and +80 mV were normalized to cell capacitance (proportional to cell size), and we plotted the average current density (CD) versus time (Fig. 3B). Under these conditions, we detected a CD of 60 pA/pF, compared to a value of 40 pA/pF in PCa cells (Holzmann *et al.*, 2015). Replacement of external Na^+^ in the bath solution by impermeable cation NMDG reversibly inhibited inward currents pointing to monovalent Na^+^ as inwardly conducted ion. This current development is absent in KO 1–5. In conclusion, the molecular basis of *I*_CAN_ in HCT116 cells is TRPM4. We did not observe any compensatory mechanisms, for example through TRPM5 in any of the HCT116 KO cells. Figure 3C shows a linear current–voltage relationship (IV) extracted from measurements in Fig. 3B. The IV exhibits a slightly positive reversal potential characteristic of TRPM4.

Overall, our results indicate that TRPM4 protein was expressed and conducted large Na^+^ currents in HCT116 cells. No compensatory mechanism was observed in patch‐clamp experiments with five different HCT116 TRPM4 knockout clones (KO 1–5).

### 3.4 Function of TRPM4 in CRC cells

We next assessed the role of TRPM4 in cell viability by performing a real‐time kinetic viability assay (Fig. 3D). After 48 h, cell viability was reduced in KO 1–5 (8–37 %) compared to normal HCT116 cells (Fig. 3E) and this decrease was significant in KO 1–2. Cell proliferation was measured using an xCELLigence^®^ RTCA DP system that allows label‐free real‐time measurements. Based on electrical impedance, a cell index reflecting cell number, size, and adhesion was determined (Dowling *et al.*, 2014) and plotted versus time (Fig. 3F). After 48 h, proliferation was decreased in KO 1–4 (10–52%) and this decrease was significant in KO 3 (Fig. 3G). KO 5 exhibited the same proliferation as HCT116. In contrast, we found no considerable difference in 5‐fluorouracil‐induced cell death between HCT116 and KO 1–5 (Fig. S3).

Migration and invasion were tested using migration plates in an xCELLigence^®^ RTCA DP system and plotted as cell index versus time (Fig. 3H–K) (Dowling *et al.*, 2014). For invasion, cell plates were precoated with Matrigel. In four of the five KO cell lines, we detected decreased migration (6–51%, Fig. 3H), which was significant after 14 h in KO 1 (Fig. 3I). Migration of KO 3 was not decreased compared to HCT116. Invasion was decreased in all five TRPM4 KO cells (14–58%, Fig. 3J,K). Overall, our findings show that TRPM4 knockout clones tend to have a lower viability and proliferation compared to HCT116. In addition, our data suggest that TRPM4 contributes to migration and invasion of CRC cells.

### 3.5 TRPM4 ion conductivity in cancer hallmark functions

**Figure 4:**
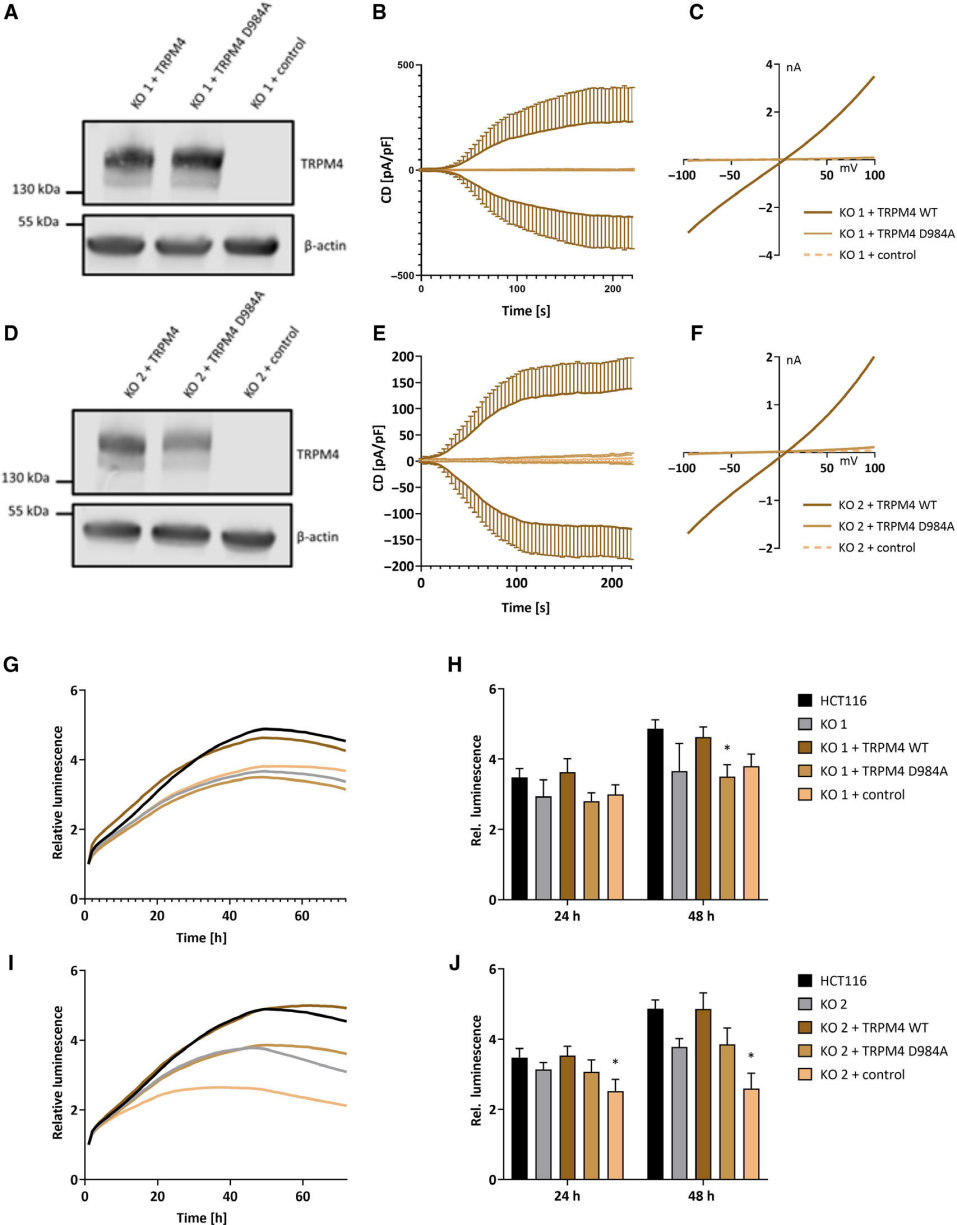
TRPM4 rescue in KO 1 and KO 2. (A) Western blot analysis of KO 1 + TRPM4 WT, KO 1 + TRPM4 D984A, and KO 1 + control (empty vector). (B) *I*_CAN_ was evoked with 10 µm Ca^2+^ in the patch pipette. Data are shown as mean ± SEM, and numbers of biologically independent replicates are *n* = 6 for KO 1 + TRPM4 WT, *n* = 4 for KO 1 + TRPM4 D984A and *n* = 8 for KO 1 + control. (C) IVs from cells in B at *t* = 196 s. (D) same as A for KO 2. (E) Same as B for KO 2. Data are shown as mean ± SEM, and numbers of biologically independent replicates are *n* = 7 for KO 2 + TRPM4 WT, *n* = 4 for KO 2 + TRPM4 D984A and *n* = 5 for KO 2 + control. (F) IVs from cells in E at *t* = 196 s. (G) Mean of cell viability in KO 1 + TRPM4 WT, KO 1 + TRPM4 D984A, and KO 1 + control. (H) Bar diagram of data (mean + SEM) at 24 and 48 h from three independent experiments in G. (I) Mean of cell viability in KO 2 + TRPM4 WT (*n* = 3), KO 2 + TRPM4 D984A (*n* = 3), and KO 2 + control (*n* = 3). (J) Bar diagram of data (mean + SEM) at 24 and 48 h from three independent experiments in I. Statistical significance was tested by a Friedman test and Dunn’s multiple comparison test. (*P < 0.05) in H and J.

Next, we investigated if cancer hallmark functions are directly dependent on TRPM4 ion channel conductivity. Functional TRPM4 (TRPM4 WT) and a nonconducting, dominant‐negative mutant of TRPM4 (TRPM4 D984A, Nilius *et al.*, 2005) were stably expressed in HCT116 TRPM4 knockout cells (KO 1–2). Re‐expression of TRPM4 in KO 1–2 was confirmed by qPCR (Fig. S4A) and western blot analysis (KO 1 in Fig. 4A and KO 2 in Fig. 4D). In both, KO 1–2 TRPM4 WT re‐expression restored TRPM4 currents, while TRPM4 KO cells expressing TRPM4 D984A failed to conduct Na^+^ (KO 1 in Fig. 4B,C, KO 2 in Fig. 4E,F).

In KO 1 and KO 2, re‐expression of functional TRPM4 reversed the tendency toward decreased viability, while expression of TRPM4 D984A did not have a rescuing effect on cell viability (KO 1 in Fig. 4G,H and KO 2 in Fig. 4I,J). Rescue experiments for other cellular functions (proliferation, migration, and invasion) were less conclusive (Fig. S4B–G).

In summary, TRPM4 contributes to cancer cell viability due to its ion conductivity.

### 3.6 TRPM4 ion conductivity in cell cycle

**Figure 5:**
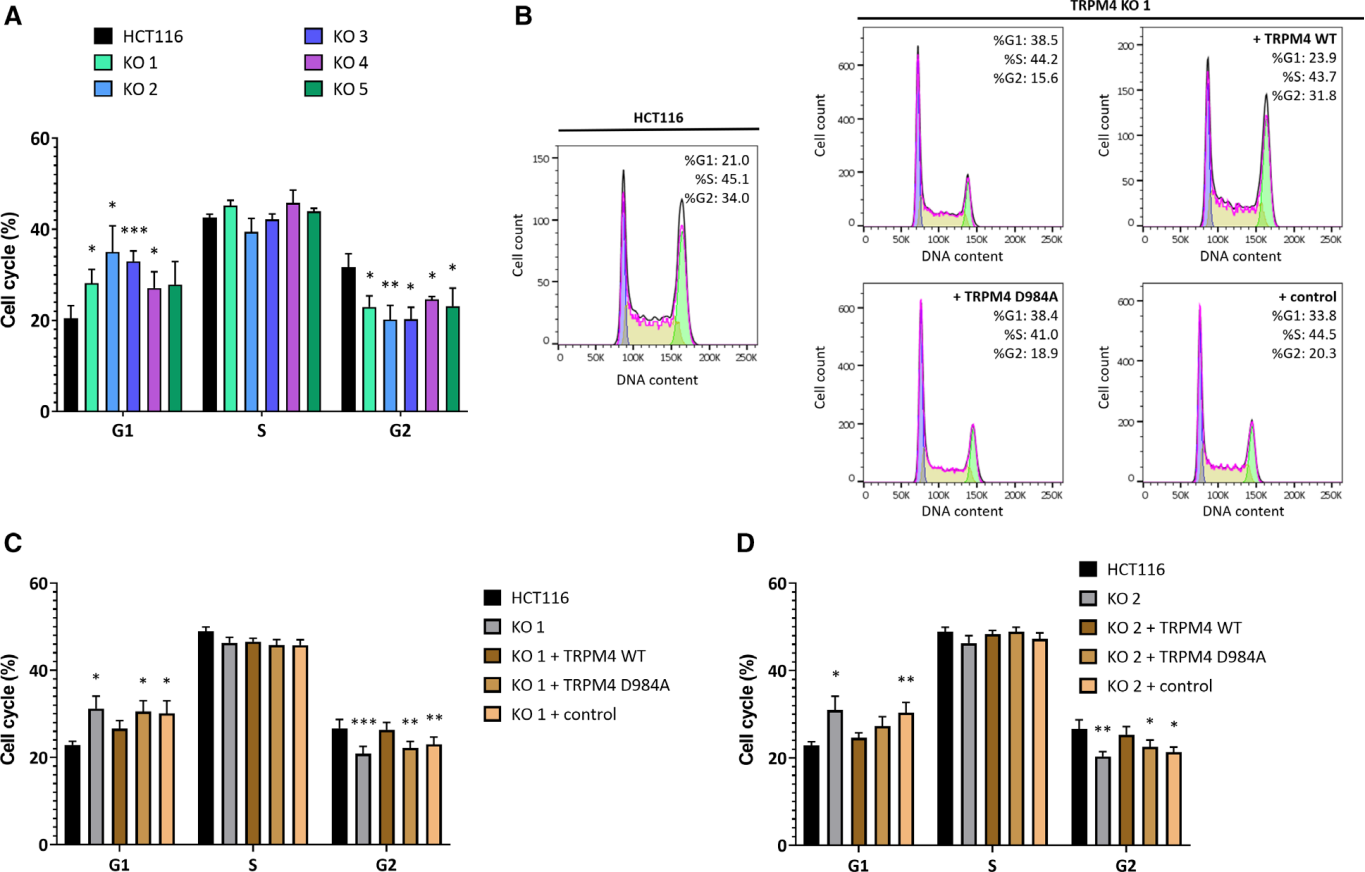
FACS‐based cell cycle analysis. (A) Bar diagram (mean + SEM) for cell cycle distribution of HCT116 and KO 1–5 from five different experiments. (B) Representative histograms of PI staining in HCT116, KO 1, KO 1 + TRPM4 D984A, and KO 1 + control. (C) Bar diagram (mean + SEM) of cell cycle distribution of HCT116, KO 1, and KO 1 + TRPM4 WT, KO 1 + TRPM4 D984A, and KO 1 + control for eight independent experiments each. (D) Bar diagram (mean + SEM) of cell cycle distribution of HCT116, KO 2, and KO 2 + TRPM4 WT, KO 2 + TRPM4 D984A, and KO 2 + control for eight independent experiments each. RM one‐way ANOVA and Holm–Sidak’s multiple comparison test were used to determine statistical significance (*P < 0.05, **P < 0.005, ***P < 0.0005) in A, C and D.

Changes in cell viability can be accompanied or caused by changes in cell cycle and TRPM4 has been reported to alter cell cycle in the past (Sagredo *et al.*, 2018). Indeed, all five TRPM4 KO cells were more likely to be in G1 phase and less likely to be in G2 phase in comparison with HCT116 (Fig. 5A, an exemplary FACS analysis is shown in Fig. 5B). These results were in line with previous work from Hong and colleagues (Hong and Yu, 2018).

Both, KO 1 and 2 with re‐expressed TRPM4 WT, showed similar cell cycle patterns as HCT116 cells (Fig. 5C,D), while expression of TRPM4 D984A failed to rescue cell cycle changes (Fig. 5C,D). In conclusion, TRPM4 ion conductivity is the underlying mechanism for TRPM4 induced changes in cell cycle.

## 4 Discussion

Our present data demonstrated that TRPM4 protein was expressed in CRC tumor tissue. Analysis of tumor features from 379 patients revealed that high levels of TRPM4 protein were related to aggressive tumor features, including high numbers of tumor buds—which is a sign of EMT, metastasis, and more aggressive infiltrative growth patterns in CRC (Fig. 1) (Georges *et al.*, 2018; Karamitopoulou *et al.*, 2015; Koelzer *et al.*, 2016). TRPM4 contributes to migration in multiple cellular systems, including immune cells, vascular endothelial cells, and PCa cells (Barbet *et al.*, 2008; Holzmann *et al.*, 2015; Sagredo *et al.*, 2019; Sarmiento *et al.*, 2015; Shimizu *et al.*, 2009). In CRC cells (HCT116), TRPM4 knockout resulted in reduced migration and invasion (Fig. 3).

In cell lines representing Dukes stages A–D, TRPM4 protein expression levels suggested that high TRPM4 protein expression levels were associated with CRC metastasis (Dukes stage D; Fig. 2). This indicates that TRPM4’s contribution to migration and invasion may be even more prominent in late‐stage CRCs. Notably, in Colo205 (Dukes stage D) TRPM4 protein levels were high, while mRNA levels were rather low. This could be due to post‐translational mechanisms that are altered in cancer cells and affect TRPM4 function, for example, glycosylation (Syam *et al.*, 2014). The imbalance of mRNA versus protein level in Colo205 may also reflect that while TRPM4 mRNA expression in CRC is decreased (Sozucan *et al.*, 2015), we find high TRPM4 protein expression correlated with tumor budding and TBC.

Overall, our data highlight a functional role of TRPM4 in tumor cell migration and invasion, which may contribute to tumor budding. This could either be due to an impairment of chemotaxis (steering mechanism) or migration (motor mechanism). Different mechanisms have been proposed for how TRPM4 contributes to cell migration, such as alteration of Ca^2+^ signaling, and alteration of the APC pathway (Cáceres *et al.*, 2015; Holzmann *et al.*, 2015; Sagredo *et al.*, 2019; Shimizu *et al.*, 2009). Alterations in the APC‐WNT/β‐catenin, transforming growth factor‐β, EGFR, and downstream MAPK and PI3K signaling pathways are nearly ubiquitous events in CRC. In ~ 80% of tumors, APC gene defects result in constitutive WNT signaling and protein expression of β‐catenin‐activated genes, such as matrix metalloproteinases (MMPs), contributing to EMT (Fearon, 2011).

SNAIL1 is a protein within the APC pathway, which suppresses synthesis of E‐cadherin, an adhesion protein that prevents EMT and promotes expression of MMPs. TRPM4 functionally contributes to EMT by regulation of SNAIL1 expression levels in PCa cells (Sagredo *et al.*, 2019). Further research is needed to investigate whether there is a coherent mechanism for TRPM4 in CRC. Notably, APC dysfunction (e.g., nuclear translocation of β‐catenin) and SNAIL1 expression have also been discussed in the context of tumor budding, as β‐catenin, SNAIL1, and MMP expression levels are elevated in tumor buds (Galván *et al.*, 2015; Jass *et al.*, 2003; Zlobec and Lugli, 2010).

Transient receptor potential melastatin‐4 knockout cells were characterized by lower viability, lower proliferation, and a cell cycle shift to G1 phase (Figs 3 and 5). These findings are in line with data from PCa cells (Hong and Yu, 2018), as TRPM4 inhibition by microRNA‐150 regulates cell cycle and proliferation *via* a cell cycle shift toward G1. Additionally, Ricardo Armisén’s group demonstrated that TRPM4 regulates cell cycle progression. Knockdown of TRPM4 results in increased degradation of β‐catenin, and a Ca^2+^‐dependent decrease in GSK‐3β activity and decreased expression of proliferation‐associated genes (Sagredo *et al.*, 2018). Notably, an siRNA‐based screening assay points to TRPM4 as a regulator of the WNT‐β catenin signaling pathway in CRC cells (Major *et al.*, 2008).

In our present study, patch‐clamp analysis demonstrated that CRC cells from Dukes stage A (HCT116) exhibited endogenous TRPM4 currents of ~ 60 pA/pF (Fig. 3). This exceeds the currents detected in other cellular systems (TRPM4 currents are ~ 40 pA/pF in PCa cells, Holzmann *et al.*, 2015). We find strong evidence that cell viability and cell cycle shift depend on TRPM4 ion conductivity (Figs 4 and 5). Future investigations will show how TRPM4 ion conductivity alters cell viability and cell cycle. Na^+^ influx *via* TRPM4 has been shown to alter Ca^2+^ signaling (Barbet *et al.*, 2008; Cheng *et al.*, 2007; Holzmann *et al.*, 2015; Kilch *et al.*, 2016; Nilius *et al.*, 2003; Shimizu *et al.*, 2009). Hence, one explanation can be that TRPM4 alters Ca^2+^ signaling important for cell viability and cell cycle in CRC. Taken together, our study finds a multifaceted, pathophysiological role for TRPM4 in CRC, highlighting TRPM4 as a putative therapeutic target in CRC therapy.

## 5 Conclusion

High TRPM4 protein expression in CRC is correlated with unfavorable tumor features (a high number of tumor buds, and a low TBC), associated with EMT, metastasis, and invasion. Investigation of cell functions in CRC cells with TRPM4 knockout suggests that TRPM4 adds to invasion—one of the initial steps to metastasis. TRPM4 ion conductivity contributes to cell viability and shifts cell cycle to G2 phase. Together with previous findings, the prominence of TRPM4 in CRC pathophysiology suggests a versatile role for TRPM4 in different types of cancer.

## Conflict of interest

The authors declare no conflict of interest.

## Author contributions

SK, CP, PS, and IZ designed the study. RB, AB, JAG, BH, SK, DRK, and PS performed experiments. BH, SK, DRK, PS, and IZ analyzed data. SK, CP, and PS wrote the manuscript.

## Supporting information


**Fig. S1**. TRPM4 antibody specificity.
**Fig. S2**. TRPM4 mRNA levels in HCT116 and TRPM4 KO 1–5.
**Fig. S3**. Apoptosis induced by 5‐FU in HCT116 and TRPM4 KO 1–5.
**Fig. S4**. Rescue experiments with selected clones KO 1 and KO 2.
**Table S1**. Patient characteristics and association with TRPM4 in the TC, TF, and TME (*n* = 379).
**Table S2**. Oligonucleotides used to generate guide RNA constructs.
**Table S3**. Primers for genotyping.
**Table S4**. Primer pairs for genotyping.
**Table S5**. Primer pairs for amplification of TRPM4 constructs.
**Table S6**. Primer pairs for amplification of TRPM4 constructs.
