# Supplementary material for: TRPM4 is highly expressed in human colorectal tumor buds and contributes to proliferation, cell cycle, and invasion of colorectal cancer cells
Source: Mol Oncol. 2019 Sep 12;13(11):2393–405. doi: 10.1002/1878-0261.12566 (PMC6822246; doi:10.1002/1878-0261.12566)
Supplement: Supplementary file 1 — Fig. S1. TRPM4 antibody specificity. Fig. S2. TRPM4 mRNA levels in HCT116 and TRPM4 KO 1–5. Fig. S3. Apoptosis induced by 5‐FU in HCT116 and TRPM4 KO 1–5. Fig. S4. Rescue experiments with selected clones KO 1 and KO 2. Table S1. Patient characteristics and association with TRPM4 in the TC, TF, and TME (n = 379). Table S2. Oligonucleotides used to generate guide RNA constructs. Table S3. Primers for genotyping. Table S4. Primer pairs for genotyping. Table S5. Primer pairs for amplification of TRPM4 constructs. Table S6. Primer pairs for amplification of TRPM4 constructs. [file MOL2-13-2393-s001.docx]

**
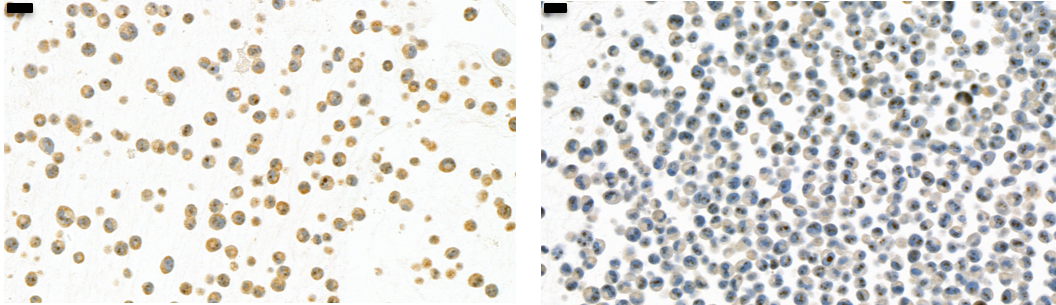
**

**Supplementary Fig. 1**: TRPM4 antibody specificity. HCT116 cells (left panel) and TRPM4 KO 5 cells (right panel) were stained using the same protocol as used for the tumor microarray, confirming the specificity of the polyclonal TRPM4 antibody. 40× magnification, 20-µm scale bar.

**
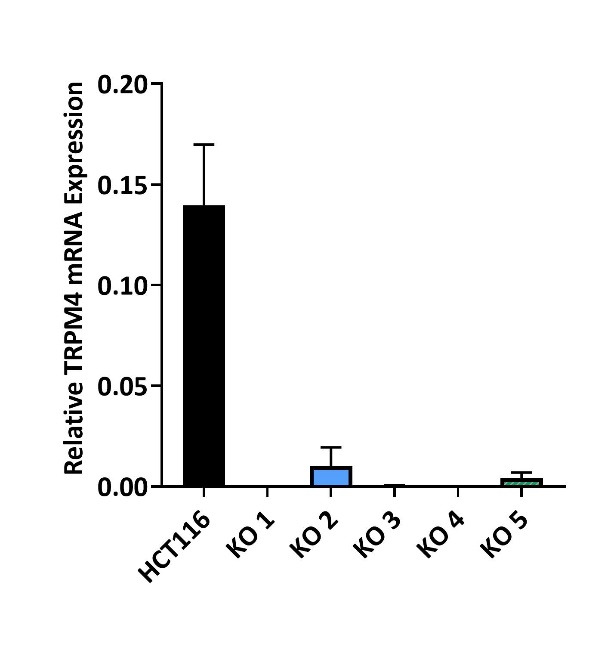
**

**Supplementary Fig. 2**: TRPM4 mRNA levels in HCT116 and TRPM4 KO 1–5. mRNA expression levels (mean + SEM) of TRPM4 were normalized to TATA-binding protein (TBP) (n = 3).

**
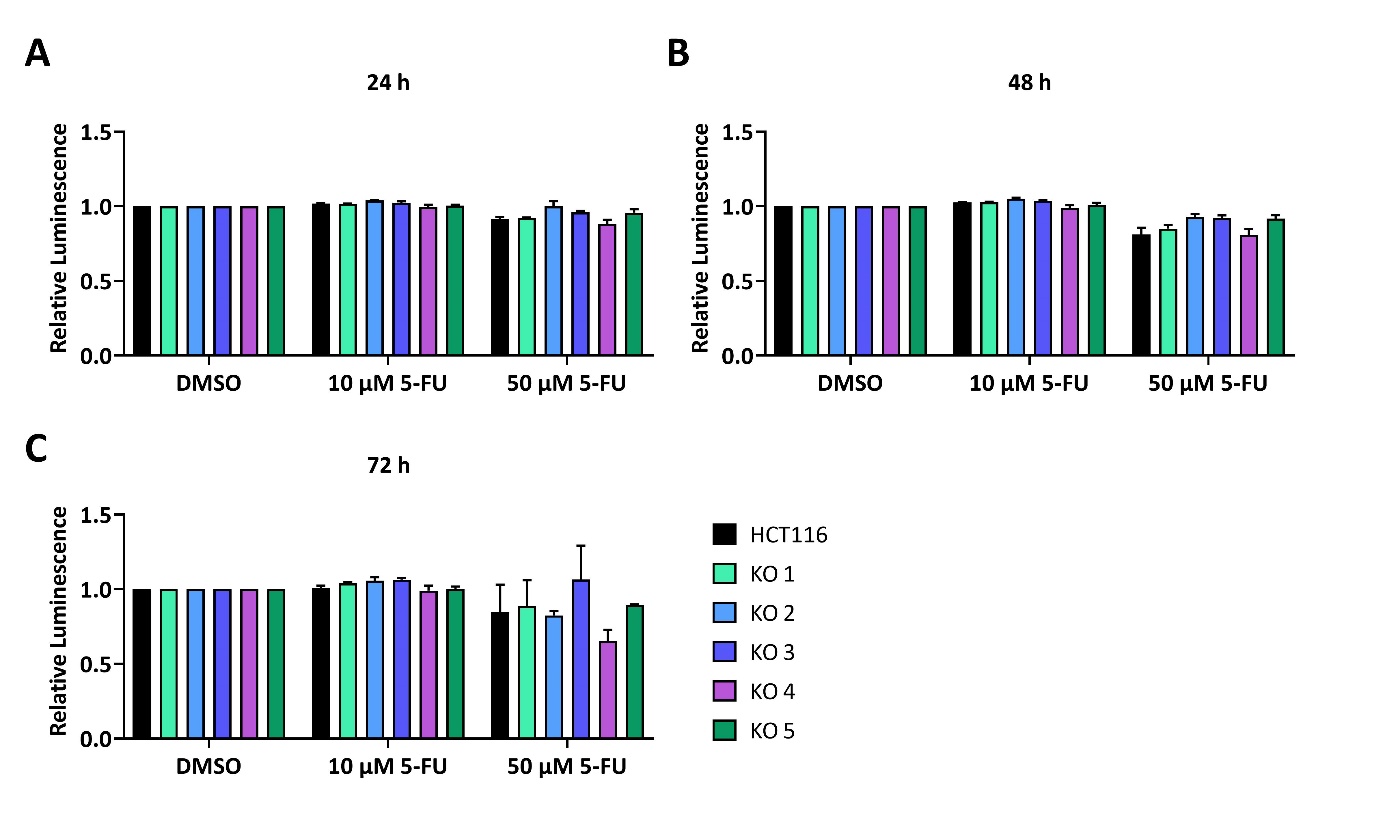
**

**Supplementary Fig. 3:** 5-FU induced apoptosis in HCT116 and TRPM4 KO 1–5. Apoptosis was induced with 10 or 50 µM 5-FU and mean + SEM of relative luminescence analyzed at 24 (**A**), 48 (**B**), and 72 hours (**C**) after treatment (n = 3).


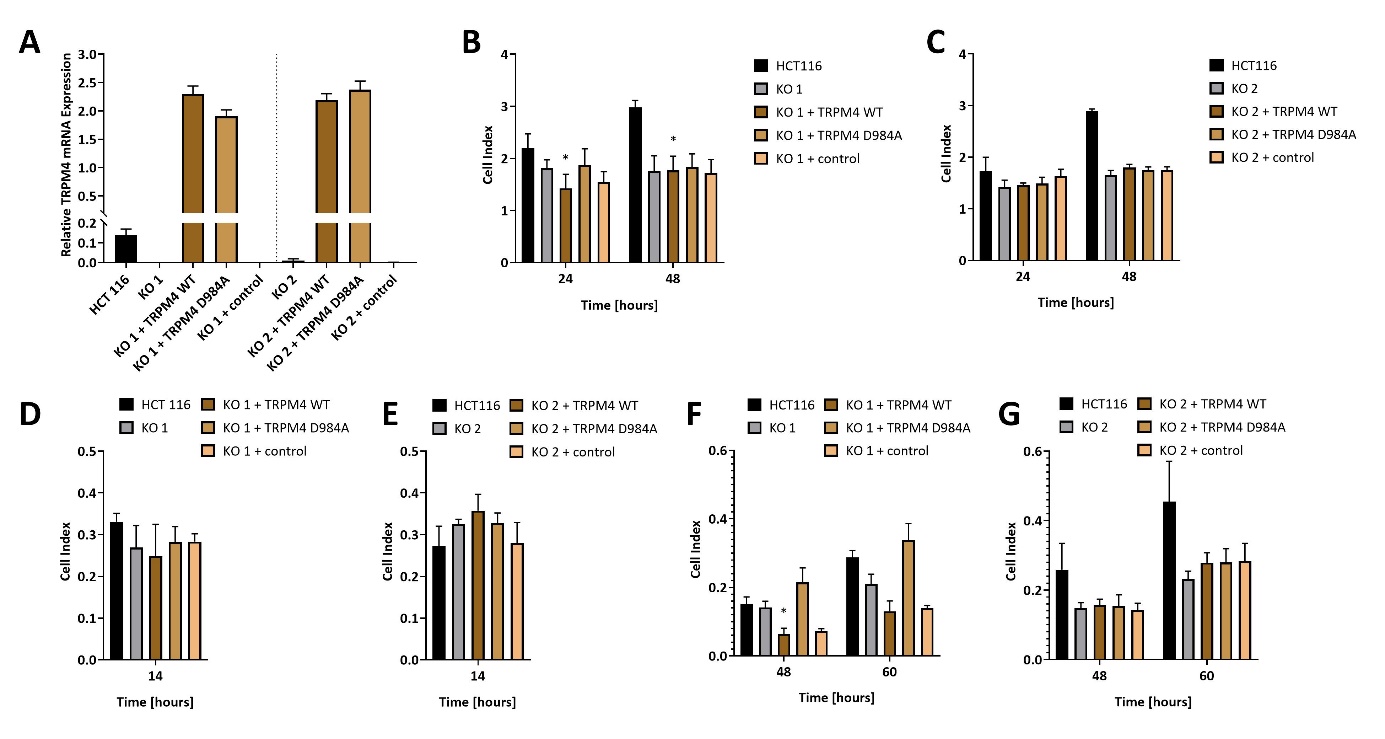


**Supplementary Fig. 4:** Rescue experiments with selected clones KO 1 and KO 2. **A** TRPM4 mRNA expression in HCT 116, KO 1 and KO 2 and after re-expression of TRPM 4 WT, TRPM4 D984A, and GFP control vector (n = 3). Proliferation (**B**+**C**), migration (**D**+**E**), and invasion (**F**+**G**) was measured at indicated time points (n = 3). Mean + SEM is displayed and statistical significance was determined by Friedman test and Dunn’s multiple comparison test.

**Supplementary Table 1** Patient characteristics and association with TRPM4 in the tumor center, tumor front, and tumor microenvironment (TME) (*n* = 379)

| Features |  | Total | TRPM4 center | | | |
| --- | --- | --- | --- | --- | --- | --- |
|  |  |  | 0 | 1 | 2 | P-value |
| Age (years) | Median, min,max | 70.6, 30-92 | 71,34-89 | 72,30-92 | 69.9,30-90 | 0.8272 |
|  |  |  |  |  |  |  |
| Tumor border configuration (% of expanding tumor border) | Median, min,max | 50, 0-100 | 50,0-100 | 50,0-100 | 40,0-100 | 0.3354 |
|  |  |  |  |  |  |  |
| Nbr. Tumor buds | Median, min,max | 7, 0-195 | 8,0-78 | 5,0-195 | 10,0-33 | 0.0144 |
|  |  |  |  |  |  |  |
| Gender | Female | 149 (39.4) | 31 | 77 | 32 | 0.535 |
|  | Male | 229 (60.6) | 37 | 116 | 55 |  |
|  |  |  |  |  |  |  |
| Histological subtype | Adenocarcinoma | 315 (87.5) | 50 | 168 | 76 | 0.0002 |
|  | Mucinous | 30 (8.3) | 7 | 14 | 4 |  |
|  | Other | 15 (4.2) | 9 | 5 | 0 |  |
|  |  |  |  |  |  |  |
| Tumor side | Left | 144 (40.6) | 29 | 83 | 26 | 0.1212 |
|  | Rectum | 90 (25.4) | 13 | 44 | 20 |  |
|  | Right | 121 (34.1) | 23 | 3 | 37 |  |
|  |  |  |  |  |  |  |
| Post-operative therapy | None | 279 (73.8) | 44 | 156 | 61 | 0.0146 |
|  | Yes | 99 (26.2) | 24 | 37 | 26 |  |
|  |  |  |  |  |  |  |
| Distant metastasis | No | 273 (722) | 53 | 134 | 66 | 0.2983 |
|  | Yes | 105 (27.8) | 15 | 59 | 21 |  |
|  |  |  |  |  |  |  |
| pT | pT1 | 7 (1.9) | 0 | 4 | 2 | 0.8571 |
|  | pT2 | 61 (16.7) | 11 | 33 | 15 |  |
|  | pT3 | 200 (54.8) | 35 | 105 | 45 |  |
|  | pT4 | 97 (26.6) | 24 | 47 | 20 |  |
|  |  |  |  |  |  |  |
| pN | pN0 | 166 (45.2) | 26 | 93 | 34 | 0.4119 |
|  | pN1 | 118 (32.2) | 25 | 62 | 26 |  |
|  | pN2 | 83 (22.6) | 16 | 35 | 22 |  |
|  |  |  |  |  |  |  |
| pTNM | I | 46 (12.6) | 7 | 27 | 11 | 0.7099 |
|  | II | 114 (31.2) | 19 | 62 | 22 |  |
|  | III | 158 (43.2) | 33 | 74 | 40 |  |
|  | IV | 48 (13.1) | 8 | 27 | 9 |  |
|  |  |  |  |  |  |  |
| Tumor grade | G1 | 7 (2.1) | 2 | 2 | 2 | 0.3154 |
|  | G2 | 256 (76.4) | 45 | 138 | 60 |  |
|  | G3 | 72 (21.5) | 18 | 36 | 11 |  |
|  |  |  |  |  |  |  |
| Lymphatic invasion | L0 | 112 (33.0) | 14 | 71 | 18 | 0.0243 |
|  | L1 | 227 (67.0) | 43 | 113 | 58 |  |
|  |  |  |  |  |  |  |
| Vessel invasion | V0 | 154 (44.9) | 23 | 83 | 35 | 0.6743 |
|  | V1 | 189 (55.1) | 36 | 102 | 41 |  |
|  |  |  |  |  |  |  |
| Perineural invasion | Pn0 | 263 (78.7) | 44 | 150 | 54 | 0.1719 |
|  | Pn1 | 71 (21.3) | 12 | 32 | 21 |  |
|  |  |  |  |  |  |  |
| Microsatellite instability status | Microsatellite instable (MSI) | 29 (14.7) | 7 | 16 | 3 | 0.212 |
|  | Microsatellite stable (MSS) | 169 (85.4) | 33 | 82 | 44 |  |
|  |  |  |  |  |  |  |
| Overall survival (OS) | 5-year survival rate (%) | 66.5% | 78.7 | 64.5 | 65.7 | 0.4311 |
|  |  |  |  |  |  |  |
| Disease-free survival (DFS) | 3-year survival rate (%) | 90.6% | 89.6 | 92.2 | 88.4 | 0.1571 |
|  |  |  |  |  |  |  |
| Features |  | Total | TRPM4 front | | | |
|  |  |  | 0 | 1 | 2 | P-value |
| Age (years) | Median, min,max | 70.6, 30-92 | 68,35-90 | 73,30-92 | 72,36-88 | 0.3556 |
|  |  |  |  |  |  |  |
| Tumor border configuration (% of expanding tumor border) | Median, min,max | 50, 0-100 | 45,0-100 | 50,0-100 | 45,0-100 | 0.3926 |
|  |  |  |  |  |  |  |
| Nbr. Tumor buds | Median, min,max | 7, 0-195 | 7,0-78 | 7,0-195 | 10,0-31 | 0.409 |
|  |  |  |  |  |  |  |
| Gender | Female | 149 (39.4) | 29 | 54 | 23 | 0.7051 |
|  | Male | 229 (60.6) | 42 | 97 | 34 |  |
|  |  |  |  |  |  |  |
| Histological subtype | Adenocarcinoma | 315 (87.5) | 59 | 129 | 48 | 0.3271 |
|  | Mucinous | 30 (8.3) | 4 | 11 | 6 |  |
|  | Other | 15 (4.2) | 4 | 4 | 0 |  |
|  |  |  |  |  |  |  |
| Tumor side | Left | 144 (40.6) | 26 | 72 | 18 | 0.1468 |
|  | Rectum | 90 (25.4) | 18 | 29 | 11 |  |
|  | Right | 121 (34.1) | 24 | 42 | 24 |  |
|  |  |  |  |  |  |  |
| Post-operative therapy | None | 279 (73.8) | 52 | 122 | 40 | 0.1969 |
|  | Yes | 99 (26.2) | 19 | 29 | 17 |  |
|  |  |  |  |  |  |  |
| Distant metastasis | No | 273 (722) | 52 | 108 | 41 | 0.9651 |
|  | Yes | 105 (27.8) | 19 | 43 | 16 |  |
|  |  |  |  |  |  |  |
| pT | pT1 | 7 (1.9) | 0 | 2 | 1 | 0.7147 |
|  | pT2 | 61 (16.7) | 12 | 24 | 7 |  |
|  | pT3 | 200 (54.8) | 35 | 85 | 33 |  |
|  | pT4 | 97 (26.6) | 23 | 35 | 14 |  |
|  |  |  |  |  |  |  |
| pN | pN0 | 166 (45.2) | 31 | 68 | 23 | 0.8509 |
|  | pN1 | 118 (32.2) | 25 | 44 | 18 |  |
|  | pN2 | 83 (22.6) | 14 | 34 | 15 |  |
|  |  |  |  |  |  |  |
| pTNM | I | 46 (12.6) | 8 | 18 | 4 | 0.9098 |
|  | II | 114 (31.2) | 22 | 48 | 17 |  |
|  | III | 158 (43.2) | 29 | 63 | 24 |  |
|  | IV | 48 (13.1) | 11 | 18 | 10 |  |
|  |  |  |  |  |  |  |
| Tumor grade | G1 | 7 (2.1) | 1 | 3 | 1 | 0.7852 |
|  | G2 | 256 (76.4) | 48 | 110 | 36 |  |
|  | G3 | 72 (21.5) | 15 | 25 | 13 |  |
|  |  |  |  |  |  |  |
| Lymphatic invasion | L0 | 112 (33.0) | 19 | 47 | 43 | 0.5256 |
|  | L1 | 227 (67.0) | 47 | 90 | 36 |  |
|  |  |  |  |  |  |  |
| Vessel invasion | V0 | 154 (44.9) | 27 | 68 | 27 | 0.3506 |
|  | V1 | 189 (55.1) | 39 | 71 | 23 |  |
|  |  |  |  |  |  |  |
| Perineural invasion | Pn0 | 263 (78.7) | 54 | 108 | 37 | 0.6031 |
|  | Pn1 | 71 (21.3) | 11 | 29 | 12 |  |
|  |  |  |  |  |  |  |
| Microsatellite instability status | Microsatellite instable (MSI) | 29 (14.7) | 7 | 8 | 5 | 0.31 |
|  | Microsatellite stable (MSS) | 169 (85.4) | 27 | 72 | 29 |  |
|  |  |  |  |  |  |  |
| Overall survival (OS) | 5-year survival rate (%) | 66.5% | 65.4 | 66.3 | 57.8 | 0.6607 |
|  |  |  |  |  |  |  |
| Disease-free survival (DFS) | 3-year survival rate (%) | 90.6% | 80.3 | 93.5 | 86.0 | 0.6399 |
|  |  |  |  |  |  |  |
| Features |  | Total | TRPM4 TME | | | |
|  |  |  | 0 | 1 | 2 | P-value |
| Age (years) | Median, min,max | 70.6, 30-92 | 67.30-88 | 73.30-92 | 70.30-90 | 0.338 |
|  |  |  |  |  |  |  |
| Tumor border configuration (% of expanding tumor border) | Median, min,max | 50, 0-100 | 50.0-100 | 50.0-100 | 30.0-100 | 0.0027 |
|  |  |  |  |  |  |  |
| Nbr. Tumor buds | Median, min,max | 7, 0-195 | 6.0-195 | 4.0-36 | 11.0-32 | 0.0005 |
|  |  |  |  |  |  |  |
| Gender | Female | 149 (39.4) | 22 | 51 | 34 | 0.3248 |
|  | Male | 229 (60.6) | 29 | 102 | 49 |  |
|  |  |  |  |  |  |  |
| Histological subtype | Adenocarcinoma | 315 (87.5) | 38 | 134 | 69 | 0.0072 |
|  | Mucinous | 30 (8.3) | 2 | 10 | 6 |  |
|  | Other | 15 (4.2) | 6 | 2 | 2 |  |
|  |  |  |  |  |  |  |
| Tumor side | Left | 144 (40.6) | 18 | 65 | 26 | 0.0512 |
|  | Rectum | 90 (25.4) | 18 | 37 | 16 |  |
|  | Right | 121 (34.1) | 14 | 47 | 35 |  |
|  |  |  |  |  |  |  |
| Post-operative therapy | None | 279 (73.8) | 34 | 121 | 58 | 0.1206 |
|  | Yes | 99 (26.2) | 17 | 32 | 25 |  |
|  |  |  |  |  |  |  |
| Distant metastasis | No | 273 (722) | 41 | 105 | 60 | 0.2687 |
|  | Yes | 105 (27.8) | 10 | 48 | 23 |  |
|  |  |  |  |  |  |  |
| pT | pT1 | 7 (1.9) | 1 | 5 | 1 | 0.8596 |
|  | pT2 | 61 (16.7) | 9 | 24 | 11 |  |
|  | pT3 | 200 (54.8) | 29 | 80 | 46 |  |
|  | pT4 | 97 (26.6) | 9 | 39 | 20 |  |
|  |  |  |  |  |  |  |
| pN | pN0 | 166 (45.2) | 18 | 73 | 32 | 0.2416 |
|  | pN1 | 118 (32.2) | 14 | 48 | 29 |  |
|  | pN2 | 83 (22.6) | 16 | 28 | 18 |  |
|  |  |  |  |  |  |  |
| pTNM | I | 46 (12.6) | 6 | 20 | 6 | 0.763 |
|  | II | 114 (31.2) | 12 | 49 | 25 |  |
|  | III | 158 (43.2) | 24 | 60 | 37 |  |
|  | IV | 48 (13.1) | 6 | 19 | 11 |  |
|  |  |  |  |  |  |  |
| Tumor grade | G1 | 7 (2.1) | 1 | 3 | 2 | 0.7735 |
|  | G2 | 256 (76.4) | 29 | 111 | 54 |  |
|  | G3 | 72 (21.5) | 11 | 25 | 16 |  |
|  |  |  |  |  |  |  |
| Lymphatic invasion | L0 | 112 (33.0) | 11 | 51 | 21 | 0.1479 |
|  | L1 | 227 (67.0) | 33 | 83 | 55 |  |
|  |  |  |  |  |  |  |
| Vessel invasion | V0 | 154 (44.9) | 16 | 61 | 37 | 0.4242 |
|  | V1 | 189 (55.1) | 28 | 77 | 39 |  |
|  |  |  |  |  |  |  |
| Perineural invasion | Pn0 | 263 (78.7) | 34 | 108 | 53 | 0.2831 |
|  | Pn1 | 71 (21.3) | 10 | 25 | 21 |  |
|  |  |  |  |  |  |  |
| Microsatellite instability status | Microsatellite instable (MSI) | 29 (14.7) | 4 | 13 | 6 | 0.6718 |
|  | Microsatellite stable (MSS) | 169 (85.4) | 17 | 61 | 43 |  |
|  |  |  |  |  |  |  |
| Overall survival (OS) | 5-year survival rate (%) | 66.5% | 66.7 | 57.2 | 73.4 | 0.0242 |
|  |  |  |  |  |  |  |
| Disease-free survival (DFS) | 3-year survival rate (%) | 90.6% | 86.9 | 92.3 | 88.3 | 0.2021 |
|  |  |  |  |  |  |  |

**Supplementary Table 2** Oligonucleotides used to generate guide RNA constructs

| **Name** | **5’-3’** | **Used for** | **Plasmid** |
| --- | --- | --- | --- |
| pBH009 | CACCGGTCAACTATGAACGTCGTGC | gRNA4.2  sense oligo | pSpCas9(BB)-2A-GFP |
| pBH010 | AAACGCACGACGTTCATAGTTGACC | gRNA4.2 antisense oligo |  |
| pBH013 | CACCGATAGTCTGGTCACACGCACA | gRNA4.1  sense oligo | pU6-(BbsI)_CBh-Cas9-T2A-mCherry |
| pBH014 | AAACTGTGCGTGTGACCAGACTATC | gRNA4.1 antisense oligo |  |
| pBH017 | CACCGTGGAGGATCCGGTTTGCTCTGGG | gRNA50fw  sense oligo | pU6-(BbsI)_CBh-Cas9-T2A-mCherry |
| pBH018 | AAACCCCAGAGCAAACCGGATCCTCCAC | gRNA50fw antisense oligo |  |
| pBH011 | CACCGAACTCCTCACCCCGGATCTG | crRNA4  sense oligo | pSpCas9(BB)-2A-GFP |
| pBH012 | AAACCAGATCCGGGGTGAGGAGTTC | crRNA4 antisense oligo |  |

**Supplementary Table 3** Primers for genotyping

| **Name** | **5’-3’** |
| --- | --- |
| pBH020 | CCTCTGTCCCCTTATCGCGG |
| pBH023 | GACGGGGAAAGACACTTAACC |
| pBH003 | ACAGTTCCTCCGGCTCTC |
| pBH042 | CTGTCTGGCATCCTCCTTTC |
| pBH002 | GAGCGCTGAGTGAGGGTTTC |

**Supplementary Table 4** Primer pairs for genotyping

| **Primer pair** | **Used for** | **Expected size in KO** | **Expected size in HCT116** |
| --- | --- | --- | --- |
| **A:** pBH020/pBH023 | Deletion 1 | 1400 bp | 12’000 bp |
| **B:** pBH023/pBH003 | Deletion 1 | No band | 950 bp |
| **C:** pBH042/pBH002 | Deletion 2 | 660 bp | 1140 bp |

**Supplementary Table 5** Primer pairs for amplification of TRPM4 constructs

| **Name** | **5’-3’** |
| --- | --- |
| pBH050 | CAAAGAATTGGGATCGCCACCATGGTGGTGCCGGAGAAGGAG |
| pBH051 | AGCGGCCGCCCTCGATTACTTGTACAGCTCGTCCA |
| pBH052 | CAAAGAATTGGGATCGCCACCATGGTGAGCAAGGGCGAGGAGCTG |

**Supplementary Table 6** Primer pairs for amplification of TRPM4 constructs

| **Primer pair** | **Used for** | **Insert size** |
| --- | --- | --- |
| **A:** pBH050/pBH51 | TRPM4 WT and TRPM4 D984A | ca. 4400bp |
| **B:** pBH052/pBH051 | GFP control | 756 bp |
